# The longitudinal mediating effect of life-space mobility on the relationship between nutritional status and cognitive function in community-dwelling older stroke patients

**DOI:** 10.3389/fpubh.2025.1677690

**Published:** 2025-10-15

**Authors:** Yixian Lei, Haixin Bai, Hongna Kang, Qi Xin, Tingting Li, Tinghui Yang, Jianan Li, Lina Meng

**Affiliations:** ^1^Harbin Medical University, Daqing Campus, Daqing, Heilongjiang, China; ^2^Key Laboratory of Basic Research and Health Management on Chronic Diseases in Heilongjiang Province, Daqing, Heilongjiang, China; ^3^Daqing Longnan Hospital, Daqing, Heilongjiang, China

**Keywords:** stroke, older adults, nutritional status, life-space mobility, cognitive function, activity

## Abstract

**Objectives:**

This study aims to explore the causal relationship between nutritional status, life-space mobility (LSM), and cognitive function in community-dwelling older stroke patients, investigate the longitudinal mediating role of life-space mobility in the relationship between nutritional status and post-stroke cognitive function.

**Methods:**

A total of 284d ischemic stroke patients aged ≥60 years were recruited from the neurology inpatient department of a tertiary hospital, with follow-up assessments conducted at 3-, 6-, and 9- months post-stroke. Mini Nutrition Assessment-Short Form (MNA-SF) were used to assess nutritional status, the Life Space Assessment (LSA-C) was used to measure life-space mobility and the global cognitive function was assessed with the Mini Mental State Examination (MMSE). Linear mixed-effects model and cross-lag-panel model was applied to examine the longitudinal relations among the variables.

**Results:**

LMM analysis revealed a significant total effect of nutritional status on post-stroke cognitive function (Estimate = 0.026, 95%CI [0.022, 0.030], *p* < 0.001). A significant indirect effect through LSM was identified (Estimate = 0.006, 95% CI [0.004, 0.008], *p* < 0.001), accounting for 24.94% of the total effect. After controlling for the longitudinal influence of LSM, the direct effect remained significant (Estimate = 0.019, 95%CI [0.015, 0.023], *p* < 0.001), indicating a partial mediating role of LSM. In contrast, the CLPM revealed a complete mediation effect for the T2-T3 pathway, with a significant indirect effect (*β* = 0.029, 95%CI [0.004, 0.061], *p* < 0.05) and a non-significant direct effect, providing complementary evidence for the mediating role of life-space mobility through a different analytical approach.

**Conclusion:**

In the chronic rehabilitation phase of older stroke patients, malnutrition can indirectly influence the development or exacerbation of post-stroke cognitive impairment via limited life-space mobility. This underscores the importance for early malnutrition identification and intervention and life-space mobility improvement to enhance long-term cognitive function in post-stroke care.

## 1 Introduction

Post-stroke cognitive impairment (PSCI) refers to objective cognitive impairment after a stroke event that persist for 3–6 months or longer. As a core complication, PSCI significantly impacts the long-term prognosis of stroke patients. It affects 24–53.4% of stroke patients, with over half experiencing impairment in one or more cognitive domains (1, 2). Notably, stroke events can advance dementia onset in the older people by approximately 4–25 years (3). This severely compromises survivors’ quality of life and survival duration while increasing societal and economic burdens. Nutritional status and life-space mobility (LSM), as modifiable factors (4–7), are strongly associated with the onset and progression of PSCI.

Patients with stroke commonly experience swallowing difficulties, leading to a significant increase in nutritional risks and the incidence of malnutrition (8). According to Havighurst’s activity theory, deteriorating nutritional status may exacerbate limited LSM and PSCI among older community-dwelling stroke patients. Malnutrition not only delays neurological recovery and activities of daily living rehabilitation but also increases the risk and severity of post-stroke complications (9). Older stroke patients exhibit a substantially higher incidence of sarcopenia than the general population due to factors like inadequate nutrient intake and reduced basal metabolic rate. This directly impairs motor functions (e.g., walking, transferring) and further restricts daily activities (10, 11). Post-stroke nutritional status is independently associated with PSCI development, particularly in the domains of overall cognitive function and attention (12). This association holds significant clinical implications. A study of hospitalized stroke patients in rehabilitation found that those at risk of malnutrition often had poorer PSCI recovery outcomes. Moreover, 75.6% of rehabilitation-phase PSCI patients had malnutrition (5). As an important indicator of older adults’ daily activity capacity and social participation, the relationship between LSM and cognitive impairment is increasingly receiving attention. The “enriched environment” theory, derived from neuroscience, suggests that complex stimulation promotes neuroplasticity and cognitive reserve (13, 14). An expanded life-space can be viewed as a form of enriched environment, providing increased physical activity, sensory input, and social engagement. A 6 years longitudinal study revealed that higher activity levels correlated positively with better cognitive function and quality of life in older adults (15). Notably, older stroke patients commonly experience markedly greater LSM restrictions than the general older community population (16–18). LSM, reflecting an individual’s movement through their environment from bedroom to beyond their town, encapsulates not only physical capacity but also the frequency of movement and the need for assistance (19). Restricted LSM in older stroke survivors signifies a loss of autonomy and is a powerful marker of diminished community participation and increased dependency. Older stroke inpatients usually opting for community-based rehabilitation, at the community level, implementing health management strategies focused on improving nutrition and enhancing LSM is essential for optimizing cognitive function and quality of life in older stroke patients (20).

The proposed relationships within our framework are supported by a growing body of empirical evidence. Firstly, the link between nutritional status and LSM is plausible. Malnutrition and sarcopenia are prevalent post-stroke and directly impair muscle strength and endurance, which are fundamental prerequisites for mobility (21, 22). Studies have shown that better nutritional status is associated with better physical performance, such as faster gait speed (23, 24), which is highly correlated with LSM. Secondly, the connection between LSM and cognitive function is well-grounded in the enriched environment theory. A broader life-space provides a multitude of cognitive stimuli (e.g., navigation, planning, encountering novel situations) and facilitates social engagement, both of which are known to promote cognitive reserve and neuroplasticity (25–27). A multitude of studies have found that higher LSM is significantly associated with better cognitive function and a lower risk of cognitive decline in older adults (28, 29).

However, while these relationships are established, the longitudinal evidence remains limited regarding the dynamic interplay among all three variables, particularly the hypothesis that LSM serves as a critical behavioral pathway mediating the influence of nutrition on cognitive outcomes after stroke. Previous studies have primarily employed cross-sectional designs, with only a limited number investigating the temporal relationship between nutritional status and post-stroke cognitive function, and the evidence linking nutritional status to LSM remains insufficient (12, 16, 30). Critically, there is a current lack of longitudinal studies dynamically tracking the synergistic effects of nutritional status and LSM on post-stroke cognitive function. Early identification of high-risk populations for PSCI and implementation of interventions are critical clinical challenges. Therefore, elucidating the temporal dynamic relationship between nutritional status, LSM, and cognitive function is particularly important.

This study is guided by an integrated conceptual framework synthesizing Havighurst’s activity theory and the enriched environment hypothesis. Activity theory posits that sustained participation in activities is fundamental to maintaining wellbeing in later life (31). In the context of stroke recovery, we posit that nutritional status is a key enabler of the physical capacity necessary for activity. The enriched environment hypothesis provides a neurobiological mechanism, stating that increased sensory, cognitive, and social stimulation promotes brain health and cognitive function (32). We conceptualize a broader life-space as a proxy for an enriched environment, offering greater opportunities for such beneficial stimulation. Within this framework, we hypothesize that: (a) poor nutritional status predisposes survivors to restricted LSM by impairing physical capacity; and (b) restricted LSM, in turn, limits environmental enrichment and social interaction, thereby hindering cognitive recovery and exacerbating cognitive decline. Thus, LSM is postulated to be a critical mediator in the pathway from nutritional status to cognitive function. This framework justifies our focus on LSM as a mediator and underscores the importance of targeting both nutrition and mobility in interventions.

Based on the concept of full-cycle rehabilitation in stroke, this study conducted longitudinal follow-ups at 3 (early subacute), 6 (late subacute), and 9 (chronic) months post-stroke (33). The study aims to explore the causal relationship between nutritional status, LSM, and post-stroke cognitive function in community-dwelling older stroke patients, test the longitudinal mediating role of LSM in the relationship between nutritional status and post-stroke cognitive function, and provide evidence supporting the optimization of rehabilitation management and intervention strategies for this population.

## 2 Methods

### 2.1 Study design

This prospective longitudinal study employed a convenience sampling method to recruit patients presenting to the neurology outpatient department of a tertiary care hospital between June 2024 and August 2024. This approach was chosen to ensure a feasible and efficient recruitment process from the available patient population during the specified study period. Participants were assessed at three time points: 3-month (T1, early subacute), 6-month (T2, late subacute), and 9-month (T3, chronic) post-stroke. This study followed the STROBE guidelines for observational studies (34).

### 2.2 Participants

The appropriate sample size was determined based on Kendall’s principle, which recommends 10 to 20 observations per variable. This study had 15 independent variables involving patients. Accordingly, the minimum required sample size ranged from 150 to 300 participants. To account for an anticipated 20% attrition rate across the three longitudinal follow-ups, the initial target sample size was inflated to a minimum of 188 to 375 participants. A total of 284 participants were included in the study (after excluding 8 dislodged at T1, 12 dislodged at T2, and 6 dislodged at T3). To account for potential selection bias due to missing follow-ups, we repeated the analyses to include only the participants who had complete data on nutritional status, LSM, and cognitive function at all three follow-ups for longitudinal analyses. A final sample of 284 participants who completed all three assessments was therefore deemed fully adequate for the planned analyses. The inclusion criteria were: (i) Meeting the 《Diagnostic criteria for ischaemic stroke in the Chinese Guidelines for the Diagnosis and Treatment of Acute Ischaemic Stroke (2018)》 and is confirmed by cranial MRI and/or CT examination; (ii) ≥ 60 years; (iii) National Institutes of Health Stroke Scale (NIHSS) score<5. The exclusion criteria were as follows: (i) with significant visual or/and hearing impairment, unable to communicate effectively and complete questionnaires; (ii) with previous history of psychiatric illness; (iii) taking central nervous system drugs (e.g., diazepam, antidepressants, etc.); and (iv) with worsening or new lesions of unstable condition.

### 2.3 Measurements

#### 2.3.1 Nutritional status

The revised version of the Mini Nutrition Assessment Short Form (MNA-SF) was used to evaluate the nutritional status of older adults (35). The MNA-SF comprises six domains: swallowing and digestion, disease stress, bed rest, mental health, and body mass index (calf circumference can be substituted) and this 14-point scale stratifies nutritional status as follows: 12–14 (normal nutritional), 8–11 (at risk of malnutrition), and 0–7 (malnourished). The MNA-SF demonstrated good internal consistency at all three timepoints (Cronbach’s *α* = 0.705, 0.717 and 0.704 respectively).

#### 2.3.2 Life-space mobility

The Life-Space Assessment (LSA) (19) is a tool that measures LSM and is also applicable in telephone interview format. The scale documents self-reported movement across environments, from confined spaces (e.g., bedrooms) to more distant places and broader regions (e.g., city or across a region), over recent 4 weeks. In addition to spatial range, the tool quantifies the frequency of arrival at each level of living space and pays particular attention to indicators of mobility independence. The final score range is 0–120, with higher scores representing greater mobility independence and <60 representing limited mobility in living space. The scale demonstrated good internal consistency at all three timepoints (Cronbach’s *α* = 0.811, 0.781 and 0.839 respectively).

#### 2.3.3 Post-stroke cognitive function

The Chinese guidelines for the diagnosis and treatment of vascular cognitive impairment (2024) recommended MMSE to distinguish non-dementia PSCI (PSCI-ND) and dementia (PSD) (Category I strongly recommended). MMSE including orientation in time and place, ability to follow simple commands, registration, attention and calculation, memory, naming, writing and figure copying, and score range from 0 to 30, with higher scores indicating better cognitive functioning (36). Cognitive function is defined as PSCI when scores were below 27. The cut–off stratified according to educational (37), PSD was considered present when the MMSE score was ≤17 for illiterate subjects, ≤20 for patients with a primary school education background, and ≤24 for those with junior high school education. The scale performed well in terms of internal consistency at all three time points (Cronbach’s *α* = 0.707, 0.722 and 0.753 respectively).

#### 2.3.4 Covariates

Demographic covariates such as age, sex, marital status, education level, health insurance, admission method, ischemic stroke classification and hospitalization duration were treated as time-invariant covariates, while chronic comorbidities (ICD-10), personal financial status, smoking and drinking considered time-varying.

### 2.4 Statistical analysis

The normality of continuous variables was assessed using the Shapiro–Wilk test and visual inspection of Q-Q plots. As the key variables (nutritional status, LSM, cognitive function) violated the normality assumption, non-parametric tests and appropriate estimators were employed in subsequent analyses. IBM SPSS Statistics (version 26.0) was utilized to analyze participant characteristics, including assessments for common method bias, descriptive statistics (expressed as frequency/percentage for categorical variables and median (IQR: P_25_, P_75_) for continuous variables), correlation analysis and the Friedman test.

To rigorously examine the longitudinal relationships and test the mediation hypothesis, we employed a linear mixed-effects modeling (LMM) framework in R using the lme4 and lmerTest packages. The data were structured in a long format to accommodate repeated measurements. Two pivotal LMMs were specified: a mediator model predicting the transformed LSM and an outcome model predicting the transformed cognitive function. Both models included fixed effects for concurrent nutritional status and time, and incorporated a random intercept for each participant to account for the non-independence of repeated measurements. The mediation role of LSM was tested using a quasi-Bayesian approach with a large number of bootstrap simulations to obtain robust estimates of the indirect effect. In order to explore temporal relationships, a cross-lagged panel model (CLPM) was implemented in Mplus 8.3, including autoregressive and cross-lagged paths, plus contemporaneous correlations. Then, we combined CLPM with post-stroke cognitive function to assess the direct and indirect effects of nutritional status and LSM on post-stroke cognitive function through mediation analyses. The post-stroke cognitive function (Tn) was regressed on the nutritional status and LSM from the preceding time waves (Tn-1). The study adopted 0.03 (small effect), 0.07 (medium effect), and 0.12 (large effect) as benchmark values for interpreting the magnitude of cross-lagged effects (38).

Linear regression tested whether nutritional status and LSM at 3 months predicted post-stroke cognitive function at 6 and 9 months using the same covariates. Two-sided *p* values <0.05 were considered statistically significant. A bias-corrected bootstrapping method with 5,000 resamples was used to assess the significance of indirect effects. Mediation was considered statistically significant if the 95% confidence interval (CI) excluded zero.

### 2.5 Ethical approval

The study protocol was approved by the Ethics Committee of the researchers’ University. Before participation, written informed consent was obtained from all participants or their legal guardians. The study was conducted in full accordance with the ethical principles of the Declaration of Helsinki.

## 3 Results

### 3.1 Common method bias

As all variables in this study were assessed using the questionnaire survey method, Harman’s single-factor test was employed to check for potential common method bias arising from the use of self-reported data. The results showed that 21 factors with eigenvalues > 1. The first factor explained 24.99% of the variance, which is below the critical threshold of 40%. This indicates that common method bias is not a significant issue in the present study.

### 3.2 Descriptive statistics

Table 1 presents the characteristics of the participants. The median age of participants was 74.0 years (IQR: 66.25–79.0). Most were male (63.4%), married (85.2%), and had a junior high or high school education (63.4%); the majority were covered by urban employee insurance (68.7%), with emergency admission as the primary mode of admission (57.7%). Table 2 reports statistical significance of longitudinal changes derived from repeated-measures analysis for key follow-up variables. MNS-SF scores, LSM, and MMSE scores in community-dwelling older stroke patients exhibit temporal stability. Participants demonstrated statistically significant enhancements in cognitive function (*p* < 0.001) and nutritional status (*p* < 0.05) across 3-, 6-, and 9-month post-stroke follow-ups (T1-T3). While LSM showed substantial gains from T1 to T3 and T2 to T3 (both *p* < 0.001), the T1-T2 interval demonstrated no significant progression (*p* = 0.28) which with LSM restriction rates maintaining stability.

**Table 1:** Descriptive analysis of participants (*N* = 284).

Variable	T1	T2	T3
Age^a,c^	74.0 (66.25, 79.0)	/	/
Sex^b,c^			
Male	180 (63.4)	/	/
Female	104 (36.6)	/	/
Marital status^b,c^			
Married	242 (85.2)	/	/
Other	42 (14.8)	/	/
Education level^b,c^			
No formal education	20 (7.0)	/	/
Primary school	62 (21.8)	/	/
Junior high school	102 (35.9)	/	/
High school/technical secondary school	78 (27.5)	/	/
Undergraduate/College	22 (7.7)	/	/
Health insurance^b,c^			
Insurance for Urban Employees	195 (68.7)	/	/
Insurance for Urban Residents	45 (15.8)	/	/
Rural Cooperative Medical	31 (10.9)	/	/
Fully Government	9 (3.2)	/	/
Out-of-Pocket	4 (1.4)	/	/
Admission method^b,c^			
Emergency admission	164 (57.7)	/	/
Outpatient admission	120 (42.3)	/	/
Ischemic stroke classification^b,c^			
Thrombotic infarction	136 (47.9)	/	/
Arterial occlusive infarction	116 (40.8)	/	/
Other specified cerebral infarction	32 (11.3)	/	/
Hospitalization duration^a,c^	6.0 (6.0, 7.0)	/	/
Chronic comorbidities^b^			
≤1	63 (22.2)	54 (19.0)	61 (21.5)
2–4	207 (72.9)	215 (75.7)	210 (73.9)
≥5	14 (5.0)	15 (5.3)	13 (4.6)
Personal financial status^b^			
Comfortable	178 (62.7)	206 (72.5)	213 (75.0)
Moderate	80 (28.2)	62 (21.8)	56 (19.7)
Strained	26 (9.2)	16 (5.6)	15 (5.3)
Smoking^b^			
No	240 (84.5)	255 (89.8)	265 (93.3)
Yes	44 (15.5)	29 (10.2)	19 (6.7)
Drinking^b^			
No	203 (71.5)	207 (72.9)	216 (76.1)
Yes	81 (28.5)	77 (27.1)	68 (23.9)
Nutritional status^b^			
Normal nutritional	128 (45.1)	145 (51.1)	185 (65.1)
At risk of malnutrition	103 (36.3)	107 (37.7)	86 (30.3)
Malnourished	53 (18.7)	32 (11.3)	13 (4.6)
LSM^b^			
Unrestricted	97 (34.2)	97 (34.2)	140 (49.3)
Restricted	187 (65.8)	187 (65.8)	144 (50.7)
Cognitive function^b^			
PSCN	58 (20.4)	117 (41.2)	127 (44.7)
PSCI-ND	151 (53.2)	104 (36.6)	103 (36.3)
PSD	75 (26.4)	63 (22.2)	54 (19.0)

^a^Continuous variable, median (IQR: P25, P75). ^b^Categorical variable, number (%).

**Table 2:** Repeated-measures analysis for nutritional status, LSM and cognitive function (*N* = 284).

Variable	T1	T2	T3	*Z*	*p*-value
Nutritional status	11.0 (8.0, 13.0)	12.0 (9.0, 13.0)	12.0 (11.0, 13.0)	−2.790	0.016 (T1-T2)
				−7.028	<0.001 (T1-T3)
				−4.238	<0.001 (T2-T3)
LSM	51.0 (34.0, 68.0)	51.75 (37.63, 68.0)	59.0 (41.13, 78.0)	−1.678	0.028 (T1-T2)
				−10.532	<0.001 (T1-T3)
				−8.853	<0.001 (T2-T3)
Cognitive function	25.0 (21.0, 26.0)	26.0 (23.0, 27.0)	26.0 (24.0, 28.0)	−9.189	<0.001 (T1-T2)
				−13.721	<0.001 (T1-T3)
				−4.532	<0.001 (T2-T3)

Values are presented as median (IQR: P_25_, P_75_); Friedman test for repeated measures across time points.

### 3.3 Correlation analysis among nutritional status, LSM and cognitive function

Spearman correlation analysis revealed that nutritional status, LSM, and cognitive function were significantly positively correlated with each other at all three time points (all *p* < 0.001), indicating stable bivariate relationships, meeting the conditions of cross-lag analysis. The detailed correlation matrix is provided in Supplementary Table S1.

### 3.4 Linear mixed-effects model (LMM) analysis

To account for within-subject correlations across all time points and provide a robust estimate of the overall mediation effect, we employed Linear Mixed-Effects Models (LMMs) (see Table 3). The LMM analysis revealed a significant total effect of nutritional status on post-stroke cognitive function (Estimate = 0.026, *p* < 0.001; 95% CI [0.022, 0.030]). Critically, a significant indirect effect through LSM was identified (Estimate = 0.006, *p* < 0.001; 95% CI [0.004, 0.008]), which accounted for 24.94% of the total effect. After controlling for this longitudinal mediating pathway via LSM, the direct effect of nutritional status remained significant (Estimate = 0.019, *p* < 0.001; 95% CI [0.015, 0.023]), indicating a partial mediating role of LSM in the overall relationship between nutritional status and cognitive function over the 9-month study period.

**Table 3:** Results of LMMs: total, direct, and indirect effects of nutritional status on post-stroke cognitive function.

	*β*	Se	*t*	*p*	95% CI		Relative Mediation effect %
					Lower 2.5%	Upper 2.5%	
Total effect	0.164	0.013	12.92	<0.001	0.140	0.189	
Direct effect	0.107	0.015	7.179	<0.001	0.078	0.136	64.92%
Indirect effect	0.058	0.017	9.17	<0.001	0.021	0.113	35.08%

Indirect effect represents the contribution of change in post-stroke cognitive function; attributable to the LSM; direct effect represents the contribution attributable to nutritional status.

### 3.5 Cross-lagged analysis for nutritional status, LSM, and cognitive function

**Figure 1:**
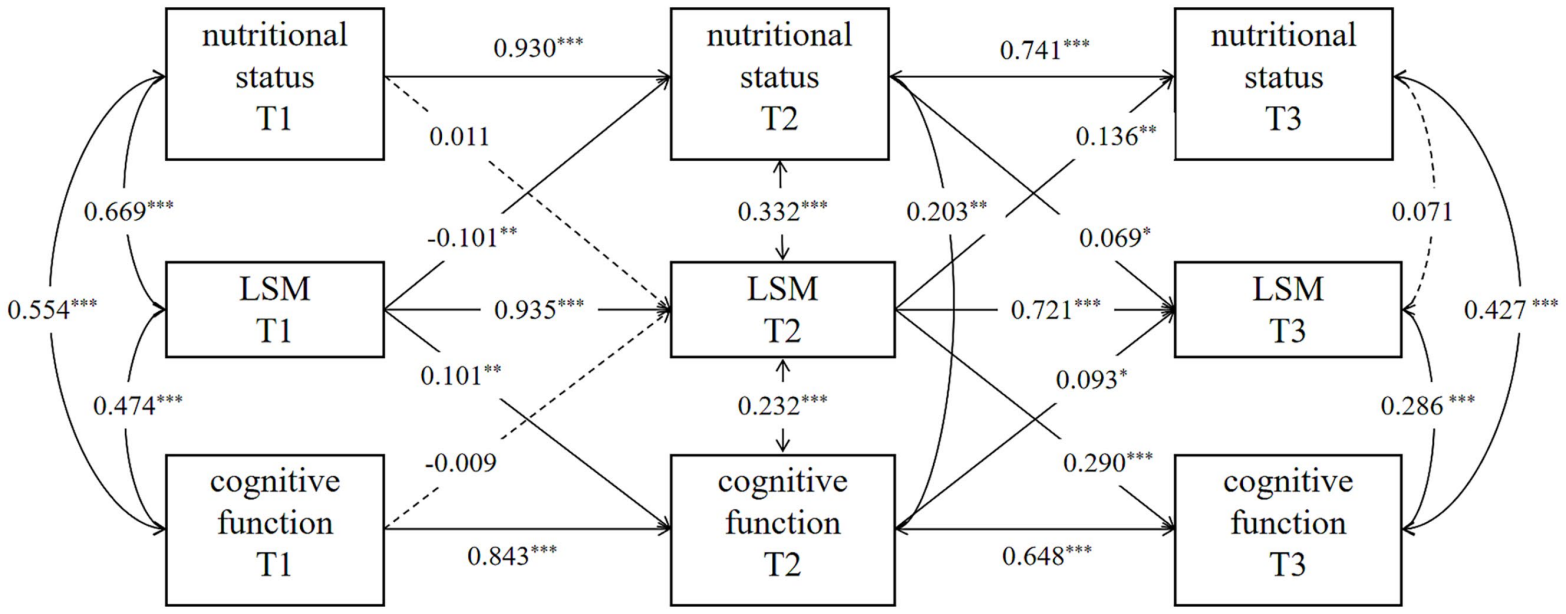
Cross-lagged panel model. Models are adjusted for age, sex, marital status, education level, health insurance, admission method, ischemic stroke classification, hospitalization duration, chronic comorbidities, personal financial status, smoking and drinking. p^*^ < 0.05, p^**^ < 0.01, p^***^ < 0.001.

To explore the lagged relationship between nutritional status, LSM, and cognitive function in community-dwelling older stroke patients, the cross-lag regression model was conducted over three assessments, as depicted in Figure 1. The autoregressive path analysis from T1 to T2 and from T2 to T3 shows that nutritional status, LSM, and cognitive function all exhibit a high degree of stability, with regression coefficients ranging from 0.741 to 0.930 for nutritional status, 0.721 to 0.935 for LSM, and 0.648 to 0.843 for cognitive function. The nutritional status of older stroke patients in the community at T2 significantly positively predicted the LSM level at T3 (*β* = 0.069, *p* < 0.05). LSM levels at T1 and T2 significantly and positively predicted cognitive function at T2 and T3, respectively (T1 → T2: β = 0.101, *p* < 0.01; T2 → T3: β = 0.290, *p* < 0.001). However, nutritional status at T1 did not predict LSM levels at T2, and neither T1 nor T2 nutritional status demonstrated significant direct effects on cognitive function at subsequent time points. These findings suggest that the LSM may mediate the relationship between nutritional status and cognitive function. The 95% confidence intervals for LSM’s mediating effects across time points are detailed in Table 4. The longitudinal mediating effect of nutritional status (T2) → cognitive function (T3) was 0.029, 95%CI [0.004, 0.061], indicating that the longitudinal mediating effect was significant, while the direct effect was not, indicating complete mediation for this specific temporal pathway (T2-T3). This result, demonstrating a complete mediation within the CLPM framework, complements the partial mediation effect identified by the LMM analysis. Together, they provide robust and multi-faceted evidence for the mediating role of LSM through different analytical approaches, with the LMM capturing the overarching partial mediation across all time points and the CLPM revealing a period of complete mediation during the chronic phase (T2-T3).

**Table 4:** LSM as a bootstrap analysis for significance testing of mediating effects.

Paths	*β*	*p*	S. E.	95%CI	
				Lower 2.5%	Upper 2.5%
Nutritional status (T1) → cognitive function (T2)	0.012	0.804	0.047	−0.078	0.106
Nutritional status (T1) → LSM (T2)	0.011	0.693	0.029	−0.044	0.071
LSM (T1) → cognitive function (T2)	0.101	0.005	0.036	0.026	0.170
Nutritional status (T2) → cognitive function (T3)	0.019	0.623	0.040	−0.095	0.058
Nutritional status (T2) → LSM (T3)	0.069	0.041	0.034	0.005	0.139
LSM (T2) → cognitive function (T3)	0.290	<0.001	0.041	0.212	0.373
Longitudinal mediating effect (T1-T3)	0.003	0.694	0.009	−0.012	0.022
Longitudinal mediating effect (T1-T2)	0.001	0.701	0.003	−0.004	0.007
Longitudinal mediating effect (T2-T3)	0.029	0.043	0.014	0.004	0.061

Mediating effect: nutritional status → LSM → cognitive function. CI: Confidence interval.

## 4 Discussion

This study employed a 9-month follow-up period to examine the developmental characteristics of the interrelationships among nutritional status, LSM, and post-stroke cognitive function in older survivors of ischemic stroke who were undergoing community-based rehabilitation. This study aimed to explore the mediating role of LSM in the relationship between nutritional status and post-stroke cognitive function for the first time. The present study employed a dual-analytic approach to unravel the longitudinal mediating role of LSM. The findings from the Linear Mixed-Effects Model (LMM) and the Cross-Lagged Panel Model (CLPM) provide complementary yet distinct insights. The LMM analysis, which accounted for within-subject correlations across all time points, indicated a significant partial mediation effect. This suggests that nutritional status influence cognitive function both directly and through its indirect effect on LSM. Thus, over the entire rehabilitation period, improving nutritional status offers broad benefits for cognition, with a substantial portion of this effect mediated by enhanced mobility and community engagement. Complementing these findings, the CLPM provided a dynamic view of temporal precedence and revealed a significant full mediation effect specifically during the transition from the late subacute to the chronic phase (T2 to T3). In this critical window, nutritional status no longer exerted a direct effect on cognitive function; instead, its influence was entirely mediated through LSM. This indicates that in the chronic recovery stage, nutritional status primarily supports cognitive function by facilitating greater LSM, which in turn may provide cognitive stimulation and social engagement. Overall, these results underscore that malnutrition and restricted LSM are interrelated risk factors that can signal heightened risk for cognitive decline. While previous longitudinal studies have identified a direct predictive relationship between nutritional status and cognitive decline or dementia (39), our advanced modeling clarifies a potential mechanism underlying this association. The findings from our dual-analytic approach suggest that the observed direct effects in earlier research may, in fact, be substantially explained by the mediating pathway of LSM, particularly in the chronic phase of stroke recovery. Additionally, age, marital status, and education level were significantly associated with post-stroke cognitive function, consistent with previous studies (40) (see Supplementary material).

Previous studies have found that in older stroke patients, malnutrition associated with both aging and post-stroke inflammatory responses, along with an increased risk of PSCI, share underlying pathological mechanisms. These involve synaptic dysfunction, neuronal death, and potential vascular pathologies (such as worsening cerebrocardiovascular disease and progression of vascular dementia), which collectively contribute to accelerated neurological decline (9, 41, 42). Although poor nutritional status may exert a fundamental influence on neuronal survival and plasticity through these pathophysiological processes, our dual-analytic longitudinal approach clarifies that the impact of nutritional status on PSCI is not monolithic but dynamic. While the LMM indicates a significant partial mediation by LSM over the entire rehabilitation period, the CLPM specifies that this mediating role becomes predominant, evolving into complete mediation, during the critical chronic phase (T2-T3). Malnutrition can affect muscle function through aberrant metabolism, leading to a negative energy balance and inducing alterations in muscle morphology and function (43, 44). The benefits of improving nutritional status largely manifest in its ability to enhance physical function (such as endurance and strength), thereby supporting daily activities and expanding participation in community activities (45). Research by Ramsey et al. (46) found that nutritional status shows a stronger association with dynamic measures of physical capacity, like walking speed, compared to static measures such as grip strength. Maintaining good nutritional status and providing nutritional support helps delay the decline in physical function and prolongs functional independence in activities of daily living (47).

LSM demonstrated a significant positive predictive relationship with post-stroke cognitive function over time. This finding not only replicates the established correlation between LSM and cognitive function observed in other populations, such as those with atherosclerotic cardiovascular disease (48), but also successfully translates it to the post-stroke context. Furthermore, it provides robust longitudinal support for the enriched environment theory. An expanded LSM, much like an enriched environment, is posited to provide the cognitive stimulation and social engagement necessary to promote neuroplasticity and cognitive reserve. Our results are consistent with prior research indicating that higher levels of physical activity and social participation are associated with better cognitive outcomes in older adults (49, 50). Based on this theory, by providing rich environmental stimuli, enhancing daily activities, and promoting social interaction, enable patients to focus limited cognitive resources on adaptive behaviors. This offers crucial behavioral protection for cognitive health, thereby aiding the recovery of post-stroke cognitive function (51, 52). This mechanism, alongside the confirmed significant longitudinal predictive effect of LSM on post-stroke cognitive function, is consistent with prior research findings. Among capable older adults, high-intensity walking activity has been shown to be significantly associated with improvements in memory (53). These findings further support the rationale for incorporating LSM into studies examining the relationship between nutritional status and cognitive function, and underscore LSM’s key bridging role in connecting nutrition to cognitive health. Crucially, LSM captures and comprehensively assesses the dynamic daily activities performed by older adults within their community.

In this study, participants exhibited a high prevalence of malnutrition, 55% were malnourished or at risk of malnutrition at 3 months post-stroke, which aligns with findings from other studies (54). Cross-sectional analyses at individual time points revealed a significant association between nutritional status and post-stroke cognitive function. Furthermore, linear regression indicated that nutritional status at T1 was significantly positively correlated with cognitive function during subsequent T2 and T3. A 3- to 5-year longitudinal study found that individuals at high risk of malnutrition were associated with a greater likelihood of cognitive decline or develop neurocognitive disorders and dementia (39). The absence of a significant direct effect of nutritional status on cognitive function in the CLPM is a key finding. This is because the CLPM controls for the autoregressive effects of cognitive function, thereby isolating the unique longitudinal effect of nutritional status on subsequent cognitive change that is not explained by prior cognitive levels or by LSM. The fact that the direct effect became non-significant after accounting for LSM underscores the latter’s critical role as a mediating pathway. The simple linear regression results, which showed a direct effect, likely captured a mixture of this mediated effect and stable between-person differences that the CLPM successfully partial out. The dynamic causal effect of nutritional status on cognitive function may not exist independently. This suggests that for older stroke patients, improving nutritional status alone may be insufficient to directly improve cognitive function, underscores the complex, multifactorial nature of PSCI and highlights the necessity for comprehensive intervention strategies.

A significant positive cross-sectional association was founded between nutritional status and LSM. This aligns with the work of Kuspinar et al. (55), who also found modifiable factors like nutrition to be related to LSM in community-dwelling older adults. Our study extends this finding by providing longitudinal evidence; the CLPM revealed that nutritional status at T2 positively predicted LSM levels at T3. This temporal precedence strengthens the plausibility of a causal relationship and suggests that improving nutritional status could actively promote greater LSM in stroke survivors, a hypothesis that warrants testing in future interventions. Notably, the CLPM indicated that neither nutritional status nor cognitive function at T1 significantly predicted LSM levels at T2 (*β* = 0.011, *p* = 0.693; β = −0.009, *p* = 0.720). This finding may be attributed to the special environmental context of the study location—winter city (56). The 6-month follow-up period (November 2024 to January 2025) coincided with the harsh winter months, characterized by persistent low temperatures and the risk of icy/slippery surfaces. Under these climatic conditions, the older population generally reduced their outdoor activities. Consequently, there was no significant difference in LSM scores between T1 and T2 (t = −0.486, *p* = 0.627), and LSM levels showed no significant improvement during this period.

An intriguing secondary finding of this study was profoundly influenced by the physical environment, particularly the seasonal winter conditions. The observed stagnation in LSM progression between T1 and T2, which coincided with the harsh winter months—stands in stark contrast to the significant improvements observed thereafter. This pattern is a powerful empirical demonstration of the “environmental press” concept within the ecological model of aging. The extended cold season in our high-latitude study location, characterized by sub-zero temperatures, pervasive ice, and reduced daylight, created a hostile mobility landscape that effectively suppressed the translation of an individual’s physiological capacity (e.g., improved nutrition, motor function) into actualized community participation and physical activities. This finding resonates with studies highlighting how environmental barriers, such as poor housing accessibility or neighborhood walkability, can significantly impact rehabilitation outcomes (57). The environmental suppression has critical implications for interpreting our longitudinal model. The non-significant path from T1 nutrition to T2 LSM likely reflects a ceiling effect imposed by winter, where the external environmental barrier masking the underlying biological relationship. Consequently, the true mediating role of LSM was only fully unmasked in the T2 to T3 period, as the thawing of spring liberated individuals to translate their recovered physiological function into expanded mobility. This suggests that the interaction between individual capacity and environmental opportunity may be an important driver of recovery. Rather than merely a confounding variable, seasonal variation represents a clinically relevant contextual factor that shapes how underlying capacity translates into daily behavior. These findings support integrating meteorological conditions and seasonal timing into both the design of rehabilitation research and the implementation of individualized, context-sensitive stroke recovery strategies.

Our findings advocate for a phased intervention strategy. While nutritional and mobility support are important throughout recovery, the transition into the chronic phase (> 6 months) emerges as a critical window where interventions must prioritize the translation of nutritional gains into expanded LSM. For instance, beyond dietary guidance, personalized plans in this phase should explicitly include graded, supported community ambulation programs, goal-setting for out-of-home activities, and occupational therapy to overcome environmental barriers. This approach directly targets the identified mechanism, LSM, through which nutrition exerts its effect on cognition in the long term.

This study has several limitations. Firstly, the use of convenience sampling from a single center may limit the generalizability of our findings. Future multi-center studies with random sampling are needed to validate our results in broader populations. Secondly, recall bias in LSM measurement, the LSM assessment relied on participants’ recall of their activity patterns over the past month, which is susceptible to recall bias. Future research could employ wearable devices (e.g., GPS trackers) to objectively monitor core dimensions such as activity space range, frequency, and independence, thereby improving measurement accuracy. Finally, sample representativeness and environmental influences: Single-center sampling resulted in a limited sample size with potentially restricted representativeness. Furthermore, the study location was situated in a winter city, where extreme low temperatures during the winter months may have led to a systematic underestimation of LSM. This environmental factor could amplify the observed differences in rehabilitation stage effects. Future studies should consider multicenter sampling across diverse regions and extend the total follow-up duration.

The findings of this study have tangible implications for practice and research. For clinical practice, our results advocate for the routine integration of dual assessments (using the MNA-SF for nutritional status and the LSA for LSM) into the standard follow-up care for community-dwelling older stroke survivors. Identifying patients with co-existing malnutrition and restricted LSM as early as 3 months post-stroke can help pinpoint individuals at the highest risk for cognitive decline. For these high-risk individuals, management strategies must move beyond siloed approaches. Multidisciplinary teams (e.g., physicians, nurses, dietitians, physical and occupational therapists) should collaborate to design personalized interventions that simultaneously target both nutritional improvement and mobility enhancement. This could include, for instance, combining dietary counseling and oral nutritional supplements with graded, supervised community ambulation programs, goal-setting for out-of-home activities, and occupational therapy focused on overcoming environmental barriers. For future research, the validated longitudinal mediating role of LSM warrants confirmation in larger, multi-center cohorts to enhance generalizability. Quantitative research into the impact of environmental factors (e.g., seasonal variations, neighborhood walkability) on LSM is crucial. Most importantly, our findings serve as a strong empirical foundation for the development and testing of novel combined interventions. We suggest that the next step be to conduct randomized controlled trials (RCTs) to evaluate whether integrated care models co-managing nutrition and mobility can effectively preserve long-term cognitive function.

## 5 Conclusion

This prospective study employed a dual-analytic longitudinal approach, utilizing both linear mixed-effects models and cross-lagged panel modeling to elucidate the dynamic relationships among nutritional status, LSM, and cognitive function in community-dwelling older stroke patients. The results demonstrate that LSM acts as a significant mediator throughout the rehabilitation period, with partial mediation observed over the full 9-month span and complete mediation specifically identified during the critical chronic phase (T2 to T3). These findings highlight that improving nutritional status can enhance cognitive outcomes both directly and indirectly through expanded life-space mobility. Consequently, healthcare professionals should adopt integrated, phase-specific intervention strategies that simultaneously target nutritional improvement and LSM enhancement to effectively prevent and manage post-stroke cognitive impairment (PSCI). Such approaches are essential for preserving long-term cognitive health and quality of life in this vulnerable population.

## Data Availability

The raw data supporting the conclusions of this article will be made available by the authors, without undue reservation.
