# Supplementary material for: The longitudinal mediating effect of life-space mobility on the relationship between nutritional status and cognitive function in community-dwelling older stroke patients
Source: Front Public Health. 2025 Oct 15;13:1677690. doi: 10.3389/fpubh.2025.1677690 (PMC12568339; doi:10.3389/fpubh.2025.1677690)
Supplement: Supplementary file 1 [file Datasheet_1.docx]

Supplementary Material

# Detailed Correlation Analysis

The results of the correlation analysis are summarized in Supplementary Table S1. The all three variables’ measurements at three time points show significant positive correlations with each other, p < 0.01. The cognitive function is significant related to nutritional status, r = 0.548 at T1, r = 0.481 at T2, r = 0.550 at T3, respectively. The cognitive function is also significant related to LSM, r = 0.437 at T1, r = 0.495 at T2, r = 0.682 at T3, respectively. Meanwhile, the nutritional status and LSM at three time points also display significant positive correlations, r = 0.684 at T1, r = 0.552 at T2, r = 0.501 at T3, respectively. Additionally, significant negative correlations are observed between nutritional status at T1 and LSM at T2, cognitive function at T2, LSM at T3, and cognitive function at T3. The two-two succeeding temporal correlation was also significant, with the previous time point of either variable being significantly correlated with the other two variable points at the subsequent time. The simultaneous correlation and stability correlation between the cognitive function and the LSM or nutritional status among older stroke patients are basically consistent, which meets the conditions of cross-lag analysis.

**Supplementary Table S1** Correlations for nutritional status, LSM and cognitive function (N = 284).

|  | | | nutritional status | | | LSM | | | cognitive function | | |
| --- | --- | --- | --- | --- | --- | --- | --- | --- | --- | --- | --- |
|  | T1 | T2 | | T3 | T1 | T2 | T3 | T1 | | T2 | T3 |
| nutritional status |  |  | |  |  |  |  |  | |  |  |
| T1 | - |  | |  |  |  |  |  | |  |  |
| T2 | 0.877^**^ | - | |  |  |  |  |  | |  |  |
| T3 | 0.736^**^ | 0.809^**^ | | - |  |  |  |  | |  |  |
| LSM |  |  | |  |  |  |  |  | |  |  |
| T1 | 0.684^**^ | 0.527^**^ | | 0.513^**^ | - |  |  |  | |  |  |
| T2 | 0.653^**^ | 0.552^**^ | | 0.548^**^ | 0.945^**^ | - |  |  | |  |  |
| T3 | 0.592^**^ | 0.503^**^ | | 0.501^**^ | 0.807^**^ | 0.834^**^ | - |  | |  |  |
| cognitive function |  |  | |  |  |  |  |  | |  |  |
| T1 | 0.548^**^ | 0.454^**^ | | 0415^**^ | 0.437^**^ | 0.405^**^ | 0.423^**^ | - | |  |  |
| T2 | 0.553^**^ | 0.481^**^ | | 0.434^**^ | 0.497^**^ | 0.495^**^ | 0.495^**^ | 0.888^**^ | | - |  |
| T3 | 0.580^**^ | 0.509^**^ | | 0.550^**^ | 0.640^**^ | 0.657^**^ | 0.682^**^ | 0.750^**^ | | 0.809^**^ | - |

p^*^ 0.05, p^**^ 0.01, p^***^ 0.001.

# Linear regression for cognitive function

To examine the association between baseline (3-month, T1) factors and future cognitive function, separate linear regression analyses were conducted (Supplementary Tables S2 and S3).

Nutritional status at T1 was significantly and positively associated with cognitive function at T2 (β = 0.430, p < 0.001) and T3 (β = 0.353, p < 0.001). LSM at T1 was significantly and positively associated with cognitive function at both T2 (β = 0.400, p < 0.001) and T3 (β = 0.462, p < 0.001). Education level was significantly associated with cognitive function at both T2 and T3. Age and marital status showed a significant association with cognitive function only at T3 while other covariates were not significant.

**Supplementary Table S2** Logistic regression analysis for MMSE at 6 and 9 months (MNA-SF).

| Characteristic | T2 | | T3 | |
| --- | --- | --- | --- | --- |
|  | β | p | β | p |
| MNA-SF | **0.430** | **<0.001** | **0.353** | **<0.001** |
| Age | -0.077 | 0.145 | **-0.141** | **0.010** |
| Sex | 0.048 | 0.298 | 0.053 | 0.231 |
| Marital status | -0.006 | 0.902 | **-0.146** | **0.020** |
| Education level | **0.342** | **<0.001** | **0.273** | **<0.001** |
| Health insurance | -0.094 | 0.059 | -0.049 | 0.398 |
| Admission method | -0.016 | 0.713 | 0.046 | 0.286 |
| Ischemic stroke classification | -0.010 | 0.831 | 0.010 | 0.830 |
| Hospitalization duration | -0.063 | 0.126 | 0.006 | 0.899 |
| Chronic comorbidities | 0.000 | 0.990 | 0.009 | 0.835 |
| Personal financial status | 0.015 | 0.718 | -0.074 | 0.105 |
| Smoking | -0.041 | 0.270 | -0.030 | 0.275 |
| Drinking | -0.050 | 0.351 | -0.094 | 0.433 |

**Supplementary Table S3** Logistic regression analysis for MMSE at 6 and 9 months (LSM).

| Characteristic | T2 | | T3 | |
| --- | --- | --- | --- | --- |
|  | β | p | β | p |
| LSM | **0.400** | **<0.001** | **0.462** | **<0.001** |
| Age | -0.052 | 0.344 | -0.064 | 0.223 |
| Sex | 0.037 | 0.431 | 0.046 | 0.265 |
| Marital status | -0.005 | 0.917 | **-0.116** | **0.038** |
| Education level | **0.407** | **<0.001** | **0.308** | **<0.001** |
| Health insurance | -0.055 | 0.276 | -0.019 | 0.707 |
| Admission method | 0.019 | 0.687 | 0.077 | 0.068 |
| Ischemic stroke classification | -0.015 | 0.752 | 0.002 | 0.965 |
| Hospitalization duration | -0.036 | 0.412 | -0.059 | 0.189 |
| Chronic comorbidities | -0.008 | 0.848 | -0.040 | 0.590 |
| Personal financial status | 0.011 | 0.792 | -0.005 | 0.900 |
| Smoking | -0.060 | 0.134 | -0.041 | 0.230 |
| Drinking | -0.037 | 0.491 | -0.045 | 0.523 |
